# Effects of dexmedetomidine at different dosages on perioperative haemodynamics and postoperative recovery quality in elderly patients undergoing hip replacement surgery under general anaesthesia: a randomized controlled trial

**DOI:** 10.1186/s13063-023-07384-z

**Published:** 2023-06-08

**Authors:** Haitong Liu, Mingjie Gao, Yongfeng Zheng, Caixia Sun, Qinyuan Lu, Donghua Shao

**Affiliations:** 1 Department of Anesthesiology, Zhenjiang First People’s Hospital, 8 Dianli Road, Zhenjiang, Jiangsu China; 2 Department of Orthopedics, Zhenjiang First People’s Hospital, 8 Dianli Road, Zhenjiang, Jiangsu China

**Keywords:** Dexmedetomidine, Haemodynamics, Recovery, Elderly, Hip replacement, General anaesthesia

## Abstract

**Background:**

Dexmedetomidine could provide some advantages to prevent postoperative complications in elderly patients undergoing under general anaesthesia. However, dexmedetomidine inhibits haemodynamics to some extent due to its sympathetic inhibition.

**Objective:**

To evaluate the effects of different doses of dexmedetomidine on haemodynamics during surgery and recovery after general anaesthesia in elderly patients undergoing hip replacement.

**Methods:**

This was a prospective randomized double-blind controlled clinical trial. Eligible patients were randomly allocated into comparative groups (normal saline (NS) and midazolam (MD), *n* = 30) and dexmedetomidine groups at different doses (D0.25/D0.5/D0.75, *n* = 30). In the D0.25/D0.5/D0.75 groups, dexmedetomidine was administered at different initial loading doses (0.25/0.5/0.75 μg/kg for 15 min) following 0.5 μg/kg/h continuous infusion until the end of the operation. In the MD group, patients were administered 0.03 mg/kg midazolam at the beginning of anaesthesia induction.

**Results:**

Compared to the MD and NS groups, there were significant decreases in MAP in the D0.5 and D0.75 groups at many time points, such as skin incision, end of operation, and from extubation until 30 min after extubation (*P* < 0.05); there were also significant decreases in HR in the D0.5 and D0.75 groups at time points including anaesthesia induction, end of operation, and from extubation to 2 h after operation (*P* < 0.05). In the D0.25 group, there were few differences in the changes in MAP and HR compared to the MD and NS groups during the entire perioperative period (*P* > 0.05). Moreover, the percentage of patients whose MAP and HR decreased > 20% of baseline was higher in the D0.75 and D0.5 groups than that in all other groups. Compared to the NS group, from the beginning to the end of the operation, the 95% confidence interval (CI) of RR for MAP below > 20% of baseline in the D0.5 and D0.75 groups was greater than 1. In particular, the CI of the RR in the D0.75 group was greater than 1 until the patient awoke from general anaesthesia (*P* < 0.05). In addition, the CI of the RR for HR below > 20% of baseline in the D0.5 group was greater than 1 compared to the NS group at the time of induction and extubation (*P* < 0.05). There was no significant difference in the possibility of developing hypotension or bradycardia in the MD or D0.25 groups compared to the NS group (*P* > 0.05). The recovery quality of patients during the post-anaesthesia period was also observed. No differences were observed among all the groups in the time to awakening or extubation after general anaesthesia (*P* > 0.05). According to the Riker Sedation-agitated Scale, dexmedetomidine significantly alleviated emergency agitation or delirium compared to NS (*P* < 0.05). In addition, the scores in the D0.5 and D0.75 groups were lower than those in the D0.25 group (*P* < 0.05).

**Conclusion:**

Dexmedetomidine could alleviate the agitation of elderly patients undergoing hip replacement after intravenous general anaesthesia combined with inhaled sevoflurane without delayed recovery. However, it is necessary to be vigilant about the haemodynamic inhibition of the drug at high dosages throughout the perioperative period. Dexmedetomidine 0.25–0.5 μg/kg as the initial loading dose followed by 0.5 μg/kg/h continuous infusion might provide comfortable recovery after general anaesthesia with slight haemodynamic inhibition.

**Trail registration:**

ClinicalTrial.gov, No. NCT05567523. Registered 05 October 2022, https://clinicaltrials.gov/ct2/show/NCT05567523?term=NCT05567523&draw=2&rank=1.

## Background

Hip fractures and femoral head necrosis are projected to become more common as the ageing population increases. Hip replacement, including partial and total hip arthroplasty, is a major surgical technique for these kinds of diseases [1]. During the perioperative period, however, elderly patients undergoing surgery are always at high risk for unstable haemodynamics, postoperative complications and mortality, especially those with multiple underlying diseases [2]. Specific and individual perioperative anaesthesia management to minimize postoperative adverse events in elderly surgical patients has always been a major concern.

Dexmedetomidine, a highly selective α2-adrenoreceptor agonist, is widely used in clinical practice for its unique characteristics, such as antianxiety and arousable sedation with minimal respiratory depression during operation or ICU stay [3, 4]. It is remarkable that dexmedetomidine has the characteristics of natural sleep-like sedation, unlike other sedatives such as propofol and etomidate [5]. In addition, the drug could also reduce pain intensity and opioid consumption without influencing the time of recovery from general anaesthesia [3]. Dexmedetomidine was shown to attenuate surgical stress and the inflammatory response during surgery, which may be beneficial for reducing perioperative complications and mortality [3, 6, 7]. A meta-analysis showed that perioperative use of dexmedetomidine significantly reduced the levels of IL-6, IL-8 and TNF-α within 24 h after surgery [8]. Moreover, dexmedetomidine protects neurons from injury induced by propofol or isoflurane during general anaesthesia [8, 9]. In addition, it has been found that dexmedetomidine could reduce the incidence of postoperative delirium (POD) in adult patients who underwent cardiac and noncardiac surgeries [4].

Midazolam is a benzodiazepine commonly used as sedative medications for surgery and general anaesthesia. It is often used in elderly patients undergoing low risk procedures due to the benefits of rapid onset, anxiolysis and haemodynamic stability compared to other sedatives. However, current anaesthetic guidelines advise the avoidance of benzodiazepines in elderly patients due to concerns of an increased risk of delirium [10]. In elderly patients, dexmedetomidine could improve sleep quality on the first postoperative night, reduce anxiety and alleviate postoperative pain [11]. Dexmedetomidine was shown to reduce the incidence of emergence agitation, POCD and POD after general anaesthesia [11–13]. This might be related to the improvement of postoperative analgesia and cerebral oxygen metabolism in patients using dexmedetomidine [14]. For elderly patients admitted to the ICU after noncardiac surgery, low-dose dexmedetomidine infusion increased survival up to 2 years and improved cognitive function and quality of life in 3-year survivors [15]. Similarly, dexmedetomidine could reduce the incidence of POD in elderly patients who underwent hip, knee or shoulder replacement under general anaesthesia, whether normal patients or patients with mild cognitive impairment [16]. Overall, the application of dexmedetomidine in surgical elderly patients under anaesthesia management could provide some advantages to prevent postoperative complications.

However, the influence of dexmedetomidine on haemodynamics should not be ignored. It has been shown that dexmedetomidine generally increases the incidence of bradycardia [11, 17, 18]. Lin showed that dexmedetomidine may result in haemodynamic compromise, including severe bradycardia and hypotension, and thus should be used very cautiously in patients with compromised brain perfusion [5]. To investigate the safety and efficacy of dexmedetomidine in healthy volunteers for postoperative sedation, two dexmedetomidine groups were designed: patients receiving infusions of either 0.2 or 0.6 μg/kg/h dexmedetomidine after a 10-min initial dose of 6 μg/kg/h dexmedetomidine [19]. The results showed that blood pressure and heart rate were significantly decreased from baseline after receiving dexmedetomidine. Ageing process and poor physical status in elderly patients often cause a higher risk of perioperative haemodynamic instability and postoperative complications, such as cardiovascular and cerebrovascular accidents, pulmonary infection and cognitive dysfunction. It is more important to maintain haemodynamic stability in elderly patients during the entire perioperative period. Unfortunately, few studies have reported the efficacy and safety of dexmedetomidine at different doses in elderly patients undergoing hip replacement surgery under general anaesthesia.

In this study, different initial doses of dexmedetomidine were used in general anaesthesia for elderly patients who underwent hip replacement to evaluate its influence on perioperative haemodynamics and postoperative recovery quality after intravenous anaesthesia combined with sevoflurane. Through the results of this study, we expected to determine the appropriate dose range of dexmedetomidine to provide satisfactory sedation and analgesia in elderly patients with stable haemodynamics during the perioperative period of hip replacement under general anaesthesia.

## Methods

### Study design and ethical approval

This was a prospective randomized double-blind controlled clinical trial. The study protocol was approved by the Medical Ethics Committee of Zhenjiang First People’s Hospital (Approval No. k-20190131-w). This study adhered to the CONSORT guidelines, and all methods were performed in accordance with the Declaration of Helsinki. The recruitment of elderly patients who underwent hip replacement began in September 2019 and ended in January 2022. All patients were approached before the operation to evaluate eligibility according to the following inclusion criteria: age ≥ 65 years; American Society of Anesthesiologists (ASA) physical status scale grade I–III; ready for hip replacement; body weight between 45 and 75 kg; body mass index (BMI) between 18 and 24 kg/m^2^; health conditions generally well according to medical history, physical examination, and laboratory tests; no signs of difficult intubation; no history of dementia or mental problems; normal cognitive function; and the ability to understand and comply with study procedures.

The patients were excluded if they met any of the following criteria:

1. Age < 65 years or > 90 years; BMI greater than 24 kg/m2; ASA grade higher than III;
2. Heart failure, severe arrhythmias, severe bradycardia (heart rate less than 40 beats/min), atrioventricular block of degree 2 or above, sick sinus syndrome, systolic blood pressure (SBP) ≥ 180 or < 90 mmHg, diastolic blood pressure (DBP) ≥ 110 or < 60 mmHg;
3. Severe liver or kidney dysfunction, severe infection, and other pathological conditions that could interfere with study results;
4. Dementia, cerebrovascular accidents within 3 months, mental illness, epilepsy and other adverse events that could interfere with study results;
5. Conditions that block communication and preoperative assessment, such as serious hearing or visual impairment; and
6. History of chronic analgesic use, long-term psychotropic medication use, alcohol or drug addiction.

### Randomization and blinding

After clinical assessment, patients who met the inclusion criteria were randomly allocated into 5 groups (*n* = 30 per group): the comparative groups (normal saline (NS) and midazolam (MD) groups) and dexmedetomidine groups at different doses (D0.25, D0.5 and D0.75 groups). Informed written consent was obtained from all patients before enrolment. The randomization was created by using the SPSS 22.0 software and sealed in envelopes. One researcher unaware of the study details opened the envelope for random numbers and prepared the study drugs before induction of anaesthesia. In the dexmedetomidine groups, the study drugs were diluted to two concentrations for later use: one diluted the drug to 15 ml for infusion before anaesthesia induction, but the total concentration of dexmedetomidine was different according to the grouping (0.25, 0.5, or 0.75 μg/kg/ml in the D0.25, D0.5, and D0.75 groups, respectively); the other diluted the same dexmedetomidine concentration (4 μg/ml) to 50 ml to maintain anaesthesia. The patients, anaesthesiologists and researchers who performed data collection and postoperative assessment were blinded to randomization and group allocation throughout the study period.

### Anaesthesia strategy

For all patients, no premedications were administered, and general anaesthesia protocols were consistent except for using different sedative drugs according to study requirements. After entering the operating room, patients were monitored continuously until the end of the operation, including electrocardiogram (ECG), arterial blood pressure, heart rate (HR), pulse oxygen saturation (SpO_2_), end-tidal carbon dioxide (EtCO_2_) and bispectral index (BIS). The vital signs were recorded automatically every 5 min for future research. Dexmedetomidine (200 μg/2 ml, Jiangsu Hengrui Medicine, Jiangsu, China) or midazolam (10 mg/2 ml, Jiangsu Enhua Medicine, Jiangsu, China) was provided and diluted with normal saline. In the dexmedetomidine and NS groups, dexmedetomidine or normal saline was infused at a rate of 60 ml/h for 15 min before anaesthesia induction and then infused continuously at a rate of 0.125 ml/kg/h to maintain anaesthesia until the end of the operation. In the MD group, patients were administered 0.03 mg/kg midazolam at the beginning of anaesthesia induction.

General anaesthesia procedures in all groups were performed as follows: patients were consecutively administered sufentanil 0.3–0.4 μg/kg, etomidate 0.15–0.2 mg/kg and rocuronium 0.6 mg/kg to induce anaesthesia. Dexamethasone (5 mg) was used during induction. Then, mechanical ventilation was provided after endotracheal intubation with a tidal volume of 8–10 ml/kg and a respiration rate of 10–12/min. During the operation, inhaled sevoflurane and intravenous remifentanil were continuously administered to maintain anaesthesia. All intravenous medications requiring continuous infusion were administered by using an infusion pump (SiluGao, GP-3100). The operation procedures were performed by three surgeons with more than 10 years of surgical experience under standard procedures. During the perioperative period, EtCO_2_ was maintained at 30–45 mmHg, SpO_2_ was maintained ≥ 95% and BIS was maintained between 40 and 60. Sufentanil 5–10 μg was administered 1 h before the end of the operation, and ondansetron 4 mg and dezocine 3–4 mg were administered 30 min before the end of the operation. After the operation, the patients were transferred into the post-anaesthesia care unit (PACU) to wait for recovery and monitored continuously until leaving the PACU, including ECG, arterial blood pressure, HR and SpO_2_. After returning to the ward, the vital signs of the patients were monitored continuously for 6 h and recorded every 1 h for future research. In addition, if the patient felt pain (VAS > 4) and required analgesia after the operation, oral diclofenac 50–75 mg would be administered. All the time points of key events were recorded, including anaesthesia induction, endotracheal intubation, skin incision, end of surgery, awakening and extubation.

Unstable haemodynamics were defined as follows: Bradycardia was defined as HR < 50 beats/min or decline > 20% of baseline. Tachycardia was defined as HR > 100 beats/min or an elevation of > 20% of baseline. Hypotension was defined as SBP < 90 mmHg or mean arterial pressure (MAP) decline > 20% of baseline. Hypertension was defined as SBP > 160 mmHg or an elevation of > 20% of baseline. Cardiovascular drugs, such as atropine, esmolol, phenylephrine, dopamine and norepinephrine, were administered to intervene in bradycardia/tachycardia or hypo/hypertension during the operation. If the patient’s condition was still unstable even with drug intervention, anaesthesiologists could choose to stop the study in case of adverse reactions.

### Data collection

The patient characteristics, including age, sex, weight, height, education, and ASA classification, were registered before the operation. In addition, the comorbidities of patients (hypertension, diabetes, anaemia, cardiac disease, etc.) were measured by the Charlson Comorbidity Index (CCI) score [15]. The primary outcomes were the changes in haemodynamics during the perioperative period, especially at the time points of key observation events, including anaesthesia induction, endotracheal intubation, skin incision, end of operation, awakening and extubation. The haemodynamic variables included SBP, DBP, MAP and HR.

Secondary outcomes were recovery time from general anaesthesia and whether waking agitation occurred. Specifically, the Riker Sedation-agitated Scale was used to evaluate delirium during recovery from general anaesthesia, and the proportion of agitation in each group was assessed according to two categories: nonagitated (levels 1–4) and agitated (levels 5–7) [20]. In addition, visual analogue scale (VAS) scores from 0 to 10 (0 = no pain at all and 10 = extreme pain) were measured to evaluate the pain intensity before and at 1, 2 and 6 h after the operation. The serum levels of haemoglobin (Hb), C-reactive protein (CRP), glucose (GLU), serum creatinine (Scr) and blood urea nitrogen (BUN) in the subjects were determined before and 24 h after the operation through laboratory examination. The serum levels of GLU were measured again at 72 h after the operation. Adverse events were also recorded, including length of hospitalization, incidences of nausea and vomiting (PONV), POD and ICU admission up to 7 days after the operation.

### Statistical analysis

The sample size calculation was based on the primary outcome of haemodynamic changes in elderly patients during operation and calculated using the PASS 15 software. According to a previous study about the decrease of heart rates after dexmedetomidine infusion in elderly patients with hip replacement under general anaesthesia [21], we selected the values of heart rates that required a larger sample size, and the sample size was calculated to be 17 (power = 0.8, *α* = 0.05). Considering a 20% dropout rate, 22 elderly patients each group were required. In our study, we enrolled 30 patients per group that greater than the expected number of subjects.

Statistical description and analysis of collected data were performed with SPSS version 22.0 (IBM Corp.). All tests were two-tailed, and *P* < 0.05 was considered statistically significant. Continuous variables with a normal distribution, including most demographic data and haemodynamic and laboratory parameters, are presented as the means ± standard deviations (SDs) and were analysed by using one-way analysis of variance (ANOVA) after Levene’s test (comparison among three or more groups). The Bonferroni test was used to control type I error for multiple testing. Skewed distribution data are presented as medians (interquartile range, IQR) and were analysed by using the independent sample Mann–Whitney *U* test or rank sum test. Categorical variables, such as sex and ASA classification, are presented as frequencies (percentages) and were analysed by using the chi-square test or Fisher’s exact test. Ranked data were analysed by using the Wilcoxon rank sum test or Kruskal–Wallis test. Spearman rank correlation analysis or linear correlation analysis was used to analyse the correlation between age or educational background and post-anaesthesia recovery or length of postoperative hospital stay. Adverse events were analysed as dichotomous variables, such as the incidences of PONV, POD and ICU admission.

## Results

### Disposition and demographics

**Fig. 1:**
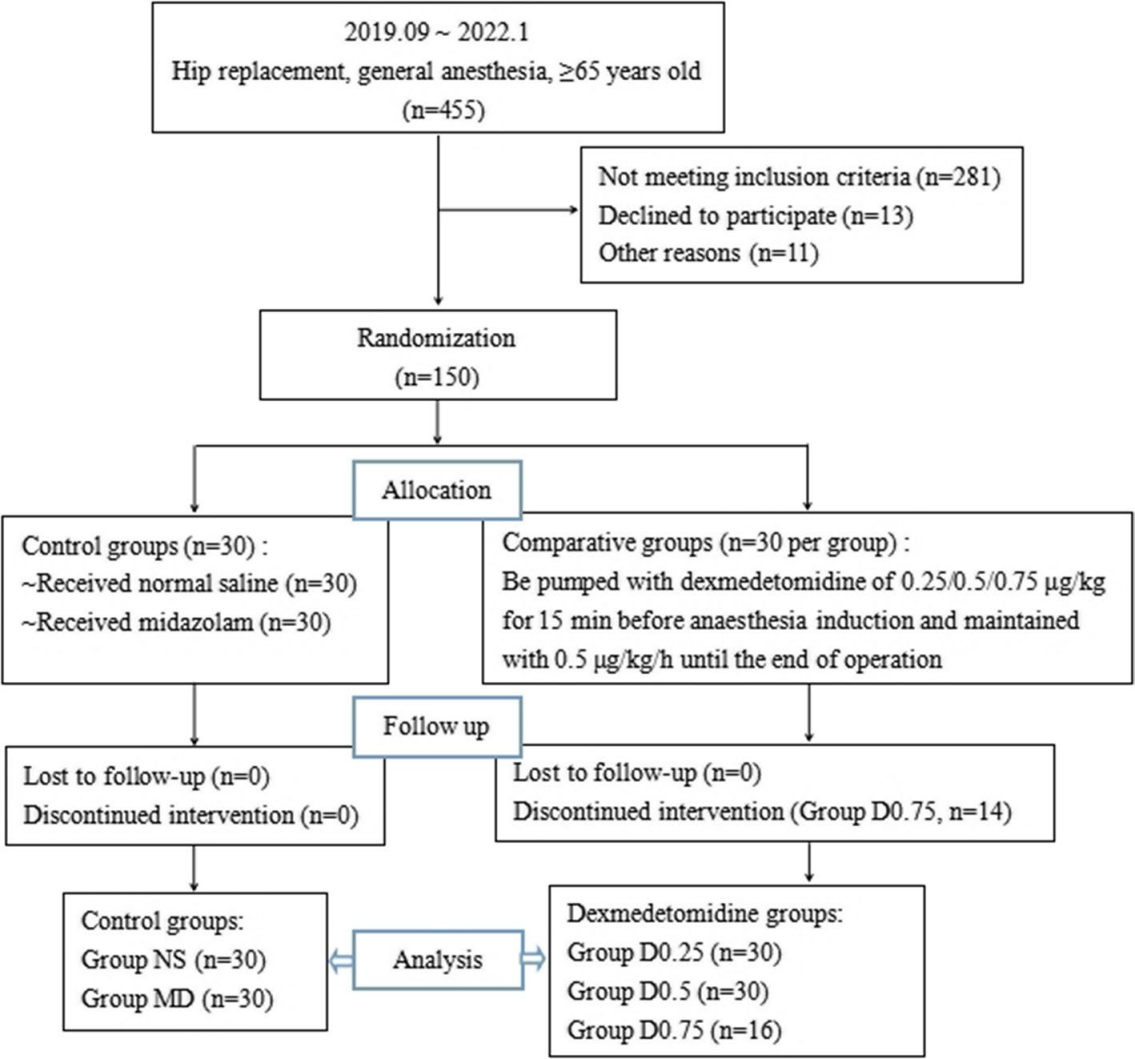
Flow chart of the study. 455 patients were recruited, and 174 patients met the inclusion criteria. Randomization was done on 150 patients, with 30 in each group. Then, 14 participants declined in the D0.75 group

The flow chart of patient enrolment is presented in Fig. 1. Between September 2019 and January 2022, 455 elderly patients who underwent hip replacement were screened, and 174 patients met the inclusion criteria and were recruited into the trial. Of the selected patients, 11 participants were excluded from the study for various reasons. In total, 150 patients were randomized into the study and allocated to the MD, NS, D0.25, D0.5 or D0.75 groups (30 per group) (Fig. 1). Fourteen patients in group D0.75 quit the study during the study procedure because their haemodynamic parameters remained so unstable (even with vasoactive drug intervention) that the continuous infusion of dexmedetomidine had to be interrupted.

### Patient characteristics

Demographic data of the subjects and surgical data are shown in Table 1. There were no significant differences in age, sex, weight, height, BMI, ASA classification, CCI scores or education among all the groups (all *P* > 0.05) (Table 1). There were more female patients than male patients in each group. For the types of patients in each group, no significant differences were found among the groups (*P* > 0.05). In addition, it should be noted that no significant differences were shown in operation time and mean length of postoperative hospital stay among the groups (*P* > 0.05). There were also no significant differences in the percentage of patients using PCIA among the groups (*P* > 0.05) (Table 1).

**Table 1:**
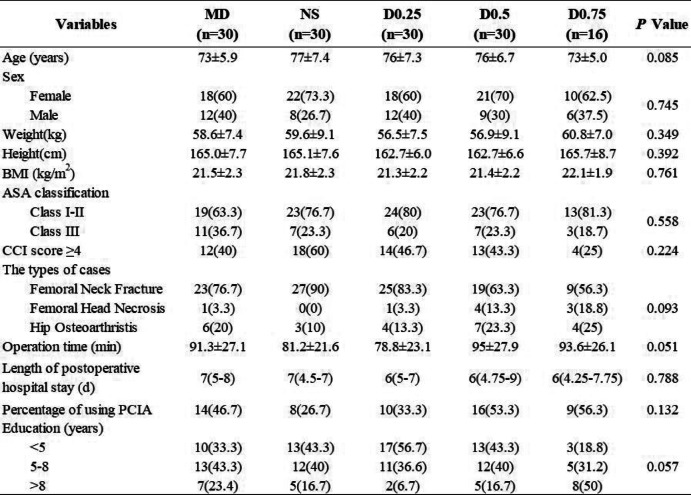
Patients characteristics

Values are expressed as mean ± standard deviation (SD), median (quartile) or *n* (%). There were no significant differences among the groups

Group MD: midazolam 0.03 mg/kg for anaesthesia induction. Group NS: normal saline 0.1 ml/kg for 15 min before anaesthesia induction + 0.125 ml/kg/h continuous infusion until the end of operation. Group D0.25: dexmedetomidine 0.25 μg/kg for 15 min before anaesthesia induction + 0.5 μg/kg/h continuous infusion until the end of operation. Group D0.5: dexmedetomidine 0.5 μg/kg for 15 min before anaesthesia induction + 0.5 μg/kg/h continuous infusion until the end of operation. Group D0.75: dexmedetomidine 0.75 μg/kg for 15 min before anaesthesia induction + 0.5 μg/kg/h continuous infusion until the end of operation

*Abbreviations*: *BMI*, body mass index; *CCI*, Charlson Comorbidity Index; *ASA*, American Society of Anesthesiologists

### Primary outcome

#### Analysis of the change in haemodynamics

**Fig. 2:**
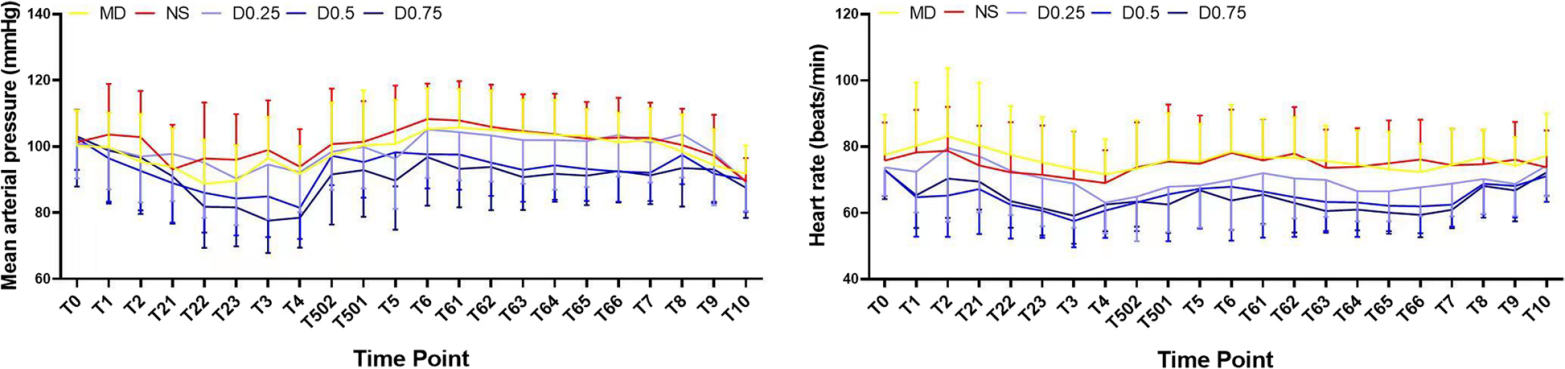
Trends of haemodynamic parameters (mean ± SD) in each group. T0: baseline, T1: start anaesthesia induction, T2: endotracheal intubation, T21, 22, 23: 5, 10, 15 min after endotracheal intubation, T3: skin incision, T4: end of operation, T502, 501: 10, 5 min before awakening, T5: awakening, T6: endotracheal extubation, T61, 62, 63, 64, 65, 66: 5, 10, 15, 20, 25, 30 min after endotracheal extubation, T7: 30 min after operation, T8, 9, 10: 1, 2, 6 h after operation. Group MD: midazolam 0.03 mg/kg for anaesthesia induction. Group NS: normal saline at the rate of 60 ml/h for 15 min before anaesthesia induction + 0.125 ml/kg/h continuous infusion until the end of operation. Group D0.25: dexmedetomidine 0.25 μg/kg for 15 min before anaesthesia induction + 0.5 μg/kg/h continuous infusion until the end of operation. Group D0.5: dexmedetomidine 0.5 μg/kg for 15 min before anaesthesia induction + 0.5 μg/kg/h continuous infusion until the end of operation. Group D0.75: dexmedetomidine 0.75 μg/kg for 15 min before anaesthesia induction + 0.5 μg/kg/h continuous infusion until the end of operation

The overall change trends of haemodynamic data in each group throughout the perioperative period are summarized in Fig. 2. There were no significant differences among the groups in terms of preoperative parameters, including MAP and HR (*P* > 0.05). After anaesthesia induction, the haemodynamic data of patients appeared to be generally decreased in the dexmedetomidine groups compared to the MD and NS groups, especially those who received a high dose of dexmedetomidine. In particular, there were significant decreases in MAP in the D0.5 and D0.75 groups at many time points, such as skin incision, end of surgery, and from extubation until 30 min after extubation, when compared to the MD and NS groups (*P* < 0.05). The heart rates were significantly lower in the D0.5 and D0.75 groups than in the MD and NS groups at time points including anaesthesia induction, end of surgery and from extubation to 2 h after surgery (*P* < 0.05). In the D0.25 group, there were few differences in MAP and HR compared to the MD and NS groups (*P* > 0.05).

**Fig. 3:**
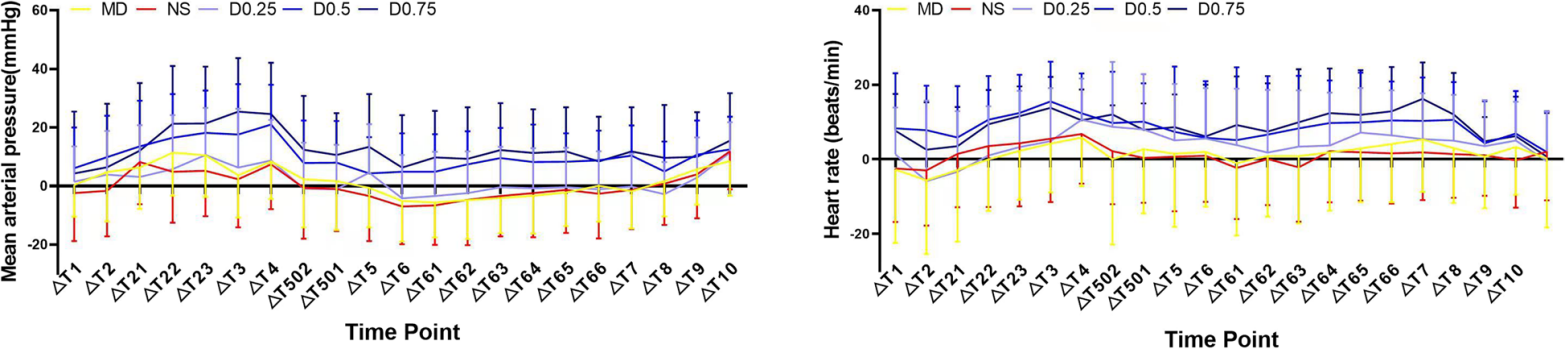
Overall fluctuation of mean arterial pressure (MAP) and heart rate (HR) compared to the baseline values in the 5 groups. Values are expressed as mean ± SD. △T1 = T0-T1, △T2 = T0-T2, △T21 = T0-T21, △T22 = T0-T22, △23 = T0-T23, △T3 = T0-T3, △T4 = T0-T4, △T502 = T0-T502, △501 = T0-T501, △T5 = T0-T5, △T6 = T0-T6, △T61 = T0-T61, △62 = T0-T62, △63 = T0-T63, △64 = T0-T64, △65 = T0-T65, △66 = T0-T66, △T7 = T0-T7, △T8 = T0-T8, △9 = T0-T9, △10 = T0-T10. T0: baseline, T1: start anaesthesia induction, T2: endotracheal intubation, T21, 22, 23: 5, 10, 15 min after endotracheal intubation, T3: skin incision, T4: end of operation, T502, 501: 10, 5 min before awakening, T5: awakening, T6: endotracheal extubation, T61, 62, 63, 64, 65, 66: 5, 10, 15, 20, 25, 30 min after endotracheal extubation, T7: 30 min after operation, T8, 9, 10: 1, 2, 6 h after operation. Group MD: midazolam 0.03 mg/kg for anaesthesia induction. Group NS: normal saline at the rate of 60 ml/h for 15 min before anaesthesia induction + 0.125 ml/kg/h continuous infusion until the end of operation. Group D0.25: dexmedetomidine 0.25 μg/kg for 15 min before anaesthesia induction + 0.5 μg/kg/h continuous infusion until the end of operation. Group D0.5: dexmedetomidine 0.5 μg/kg for 15 min before anaesthesia induction + 0.5 μg/kg/h continuous infusion until the end of operation. Group D0.75: dexmedetomidine 0.75 μg/kg for 15 min before anaesthesia induction + 0.5 μg/kg/h continuous infusion until the end of operation

The overall fluctuation of haemodynamic data compared to the baseline values in each group throughout the perioperative period is summarized in Fig. 3. We found that compared with the MD and NS groups, the higher dose dexmedetomidine groups had higher incidences of low MAP and HR. MAP was generally lowest in the D0.75 group among all the groups throughout the perioperative period, especially from skin incision to 30 min after surgery, compared to the NS group (*P* < 0.05). In addition, the HR was also generally lowest in the D0.5 and D0.75 groups throughout the operation and even until 1 h after the operation. Overall, a higher dose of dexmedetomidine may lead to a greater decrease in haemodynamic parameters in elderly patients undergoing hip replacement under general anaesthesia.

#### Analysis of decreases > 20% of haemodynamic baseline

We then evaluated the percentage of patients whose MAP and HR decreased > 20% of baseline in each group (Table 2). It was shown that the proportion of patients with hypotension and bradycardia appeared higher in the D0.5 and D0.75 groups than those in the other groups. For example, when the operation began (skin incision), the percentages of hypotension in the MD, NS, D0.25, D0.5 and D0.75 groups were 13.3%, 16.7%, 33%, 43.3% and 68.8%, respectively; the percentages of bradycardia in the MD, NS, D0.25, D0.5 and D0.75 groups were 10%, 30%, 23.3%, 53.3% and 43.8%, respectively.

**Table 2:**
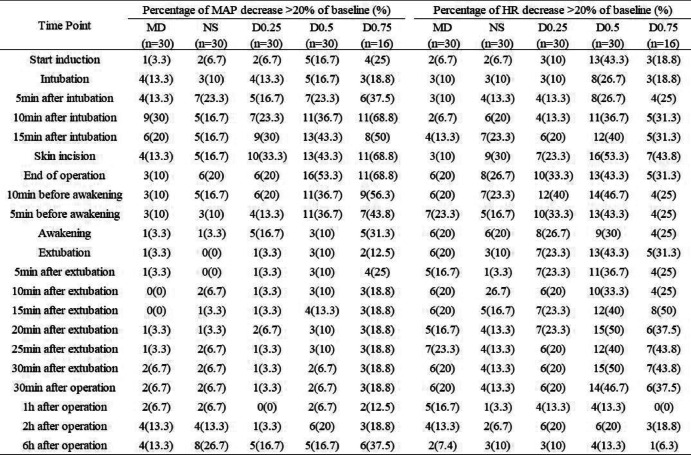
Percentage of mean arterial pressure (MAP) and heart rate (HR) decrease > 20% of baseline in the 5 groups. Values are expressed as No. (%)

Group MD: midazolam 0.03 mg/kg for anaesthesia induction. Group NS: normal saline at the rate of 60 ml/h for 15 min before anaesthesia induction + 0.125 ml/kg/h continuous infusion until the end of operation. Group D0.25: dexmedetomidine 0.25 μg/kg for 15 min before anaesthesia induction + 0.5 μg/kg/h continuous infusion until the end of operation. Group D0.5: dexmedetomidine 0.5 μg/kg for 15 min before anaesthesia induction + 0.5 μg/kg/h continuous infusion until the end of operation. Group D0.75: dexmedetomidine 0.75 μg/kg for 15 min before anaesthesia induction + 0.5 μg/kg/h continuous infusion until the end of operation

We also analysed the relative risk (RR) for MAP and HR below > 20% of baseline between the MD, D0.25, D0.5 and D0.75 groups and the NS group (Table 3). Compared to the NS group, from the beginning of the operation (skin incision) to the end of the operation, the 95% confidence interval (CI) of RR for MAP below > 20% of baseline in the D0.5 and D0.75 groups was greater than 1. Moreover, the CI of RR in the D0.75 group was greater than 1 until the patient awoke from general anaesthesia (*P* < 0.05). These results indicated that during the entire perioperative period, patients in the D0.5 and D0.75 groups were more likely to develop hypotension than those in the NS group, especially those in the D0.75 group. The duration of significant hypotension could be consecutive or nonconsecutive. The RR of hypotension between the D0.25 and MD groups and the NS group was less than 1, indicating that there was no significant difference in the possibility of developing hypotension in the D0.25 and MD groups compared to the NS group (*P* > 0.05). Similarly, according to the RR for HR below > 20% of baseline, the patients in the D0.5 group were more likely to develop bradycardia than those in the NS group at the time of induction and extubation (*P* < 0.05). The duration of significant bradycardia could be consecutive or nonconsecutive. There was no significant difference in the possibility of developing bradycardia in the MD, D0.25 and D0.75 groups compared to the NS group (*P* > 0.05).

**Table 3:**
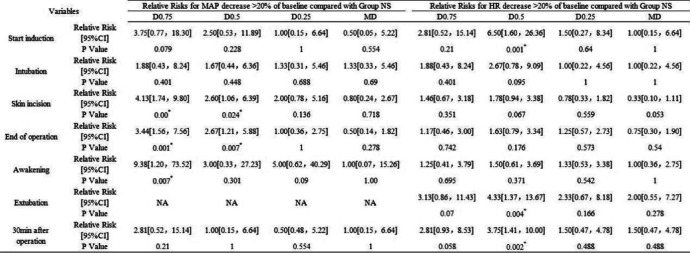
Relative risks for mean arterial pressure (MAP) and heart rate (HR) decrease > 20% of baseline compared with Group NS

Group MD: midazolam 0.03 mg/kg for anaesthesia induction. Group NS: normal saline at the rate of 60 ml/h for 15 min before anaesthesia induction + 0.125 ml/kg/h continuous infusion until the end of operation. Group D0.25: dexmedetomidine 0.25 μg/kg for 15 min before anaesthesia induction + 0.5 μg/kg/h continuous infusion until the end of operation. Group D0.5: dexmedetomidine 0.5 μg/kg for 15 min before anaesthesia induction + 0.5 μg/kg/h continuous infusion until the end of operation. Group D0.75: dexmedetomidine 0.75 μg/kg for 15 min before anaesthesia induction + 0.5 μg/kg/h continuous infusion until the end of operation

*Statistically significant (*P* < 0.05)

### Secondary outcomes

#### Recovery stage after anaesthesia

As an important secondary outcome, the recovery quality of patients during the post-anaesthesia period is shown in Table 4. No differences were observed among all the groups in the time to awakening or extubation after general anaesthesia (*P* > 0.05). We observed the effect of dexmedetomidine on patient agitation during the recovery period from general anaesthesia. Compared to the NS group, dexmedetomidine could significantly alleviate emergency agitation or delirium (*P* < 0.05); moreover, the Riker Sedation-agitated Scale scores in the D0.5 and D0.75 groups were lower than those in the D0.25 group (*P* < 0.05).

**Table 4:**
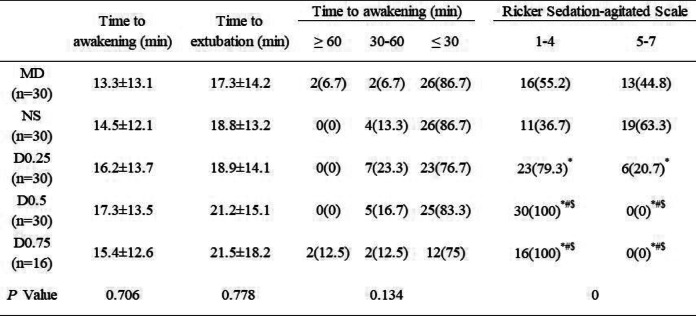
Post-anaesthesia outcomes. Values are expressed as mean ± SD or *n* (%)

Group MD: midazolam 0.03 mg/kg for anaesthesia induction. Group NS: normal saline at the rate of 60 ml/h for 15 min before anaesthesia induction + 0.125 ml/kg/h continuous infusion until the end of operation. Group D0.25: dexmedetomidine 0.25 μg/kg for 15 min before anaesthesia induction + 0.5 μg/kg/h continuous infusion until the end of operation. Group D0.5: dexmedetomidine 0.5 μg/kg for 15 min before anaesthesia induction + 0.5 μg/kg/h continuous infusion until the end of operation. Group D0.75: dexmedetomidine 0.75 μg/kg for 15 min before anaesthesia induction + 0.5 μg/kg/h continuous infusion until the end of operation

^*^P < 0.05 vs group NS

^#^*P* < 0.05 vs group MD

^$^*P* < 0.05 vs group D0.25

#### Postoperative VAS pain score

As shown in Table 5, the postoperative pain conditions for different groups were determined by VAS score. At 2 h and 6 h after the operation, the scores of patients in all the dexmedetomidine groups were significantly lower than those of patients in the NS group (*P* < 0.05). Patients in the D0.75 group appeared to have lower VAS scores.

**Table 5:**
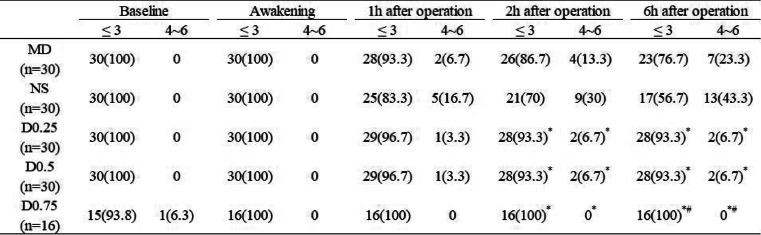
VAS scores in the different groups. Values are expressed as *n* (%)

*VAS*, visual analogue scale

Group MD: midazolam 0.03 mg/kg for anaesthesia induction. Group NS: normal saline at the rate of 60 ml/h for 15 min before anaesthesia induction + 0.125 ml/kg/h continuous infusion until the end of operation. Group D0.25: dexmedetomidine 0.25 μg/kg for 15 min before anaesthesia induction + 0.5 μg/kg/h continuous infusion until the end of operation. Group D0.5: dexmedetomidine 0.5 μg/kg for 15 min before anaesthesia induction + 0.5 μg/kg/h continuous infusion until the end of operation. Group D0.75: dexmedetomidine 0.75 μg/kg for 15 min before anaesthesia induction + 0.5 μg/kg/h continuous infusion until the end of operation

^*^P < 0.05 vs group NS

^#^*P* < 0.05 vs group MD

^$^*P* < 0.05 vs group D0.25

#### Laboratory parameters

The clinical laboratory parameters of the patients are shown in Table 6. We compared the serum levels of haemoglobin (Hb), C-reactive protein (CRP), glucose (GLU), serum creatinine (Scr) and blood urea nitrogen (BUN) of the patients in different groups before and 24 h after the operation. No significant differences were found in any of these markers before or 24 h after the operation (all *P* > 0.05).

**Table 6:**
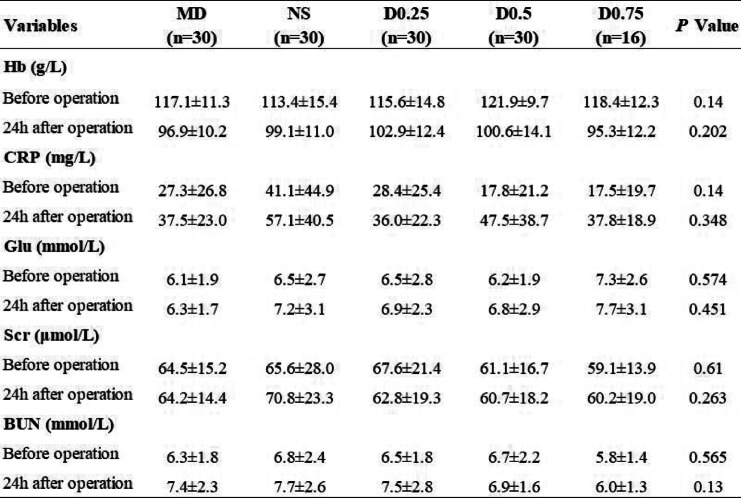
Laboratory information in different groups

Values are presented as mean ± SD. There were no significant differences among the groups

*Hb*, haemoglobin; *CRP*, C-reactive protein; *Glu*, glucose; *Scr*, serum creatinine; *BUN*, blood urea nitrogen

Group MD: midazolam 0.03 mg/kg for anaesthesia induction. Group NS: normal saline at the rate of 60 ml/h for 15 min before anaesthesia induction + 0.125 ml/kg/h continuous infusion until the end of operation. Group D0.25: dexmedetomidine 0.25 μg/kg for 15 min before anaesthesia induction + 0.5 μg/kg/h continuous infusion until the end of operation. Group D0.5: dexmedetomidine 0.5 μg/kg for 15 min before anaesthesia induction + 0.5 μg/kg/h continuous infusion until the end of operation. Group D0.75: dexmedetomidine 0.75 μg/kg for 15 min before anaesthesia induction + 0.5 μg/kg/h continuous infusion until the end of operation

#### Adverse events

All patients had uneventful procedures under general anaesthesia. The common adverse events during the perioperative period were related to PONV, POD, delayed recovery and respiratory or circulatory complications that needed to be treated in the ICU. Postoperative adverse events in the 5 groups are recorded in Table 7. The results showed that the incidences of adverse events were generally similar among all the groups, including POCD, POD and ICU admission (all *P* > 0.05). No severe side effects were observed.

**Table 7:**
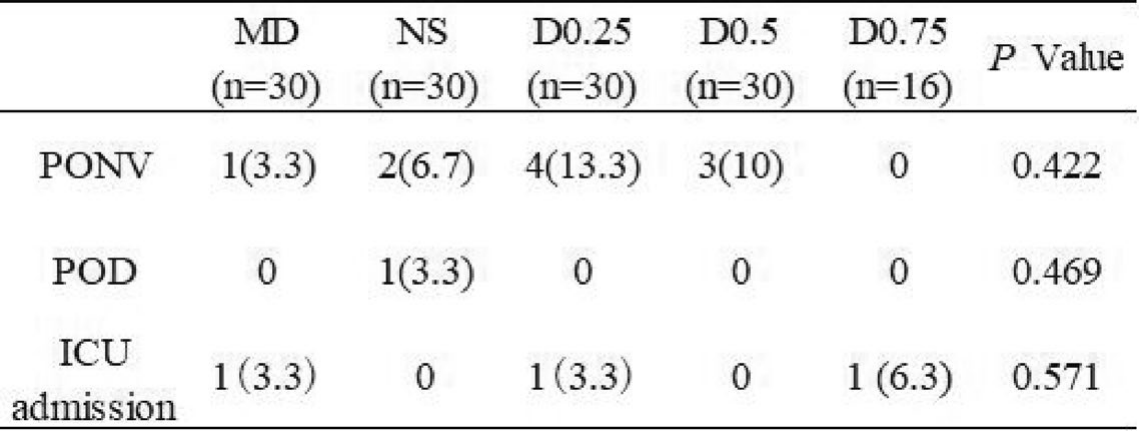
Incidence of PONV, POD and ICU admission in different groups

Values are presented as *n* (%). There were no significant differences among the groups

*PONV*, postoperative nausea and vomiting; *POD*, post delirium; *ICU*, intensive care unit

Group MD: midazolam 0.03 mg/kg for anaesthesia induction. Group NS: normal saline at the rate of 60 ml/h for 15 min before anaesthesia induction + 0.125 ml/kg/h continuous infusion until the end of operation. Group D0.25: dexmedetomidine 0.25 μg/kg for 15 min before anaesthesia induction + 0.5 μg/kg/h continuous infusion until the end of operation. Group D0.5: dexmedetomidine 0.5 μg/kg for 15 min before anaesthesia induction + 0.5 μg/kg/h continuous infusion until the end of operation. Group D0.75: dexmedetomidine 0.75 μg/kg for 15 min before anaesthesia induction + 0.5 μg/kg/h continuous infusion until the end of operation

## Discussion

In this study, a prospective randomized double-blind controlled clinical trial was performed on elderly patients who underwent hip replacement surgery under intravenous general anaesthesia combined with inhaled sevoflurane. We compared the influences of dexmedetomidine at different initial doses and midazolam on perioperative haemodynamics and recovery quality after general anaesthesia. The results showed that dexmedetomidine could reduce cardiovascular responses caused by surgical stress throughout the perioperative period, such as endotracheal intubation, skin incision and extubation. However, higher loading doses of dexmedetomidine (D0.5 and D0.75 groups) were more likely to lead to hypotension and bradycardia. On the other hand, the higher doses of dexmedetomidine (D0.5 and D0.75 groups) seemed to reduce agitation and improve tolerance to endotracheal catheters after general anaesthesia without delayed recovery.

Surgical stimuli activate the sympathetic nervous system and initiate a cascade of stress responses [17]. The stimulation of immunological and inflammatory reactions increases the secretion of cytokines and disturbs the normal proinflammatory and anti-inflammatory cytokine balance, which can increase morbidity and mortality. Attenuation of sympathetic activation to reduce the stress response during surgery has been shown to be beneficial for postoperative outcomes [17]. A meta-analysis with high-quality evidence showed that dexmedetomidine could improve subendocardial blood flow and reduce stress responses and pain by inhibiting the sympathetic nervous system [22]. Comparable to epidural anaesthesia, intraoperative infusion of dexmedetomidine attenuates surgical stress when combined with total intravenous general anaesthesia [6]. In detail, perioperative administration of dexmedetomidine could inhibit the concentrations of epinephrine, norepinephrine, cortisol and blood glucose, thereby alleviating the surgical stress response [18, 22]. However, it should be noted that dexmedetomidine also inhibits haemodynamics to some extent due to its sympathetic inhibition.

Individualized intra- and postoperative anaesthesia management to maintain haemodynamic stabilization, control effective postoperative pain and prevent hypoxemia will reduce postoperative adverse events in the elderly [2, 23]. In addition, slowing HR appropriately could reduce myocardial oxygen consumption and increase coronary artery perfusion, which is beneficial to provide cardiac protection for elderly patients during the perioperative period. However, excessive blood pressure inhibition is a danger signal for elderly individuals, which may lead to cardiovascular and cerebrovascular accidents. Inappropriate administration of dexmedetomidine may increase the risk of undesirable postoperative complications in elderly patients. Researchers studied the intranasal administration of dexmedetomidine to assess its safety and suitability in procedural sedation of the growing group of vulnerable elderly patients. The results showed that intranasal dexmedetomidine in elderly subjects had a sedative effect but caused a high incidence of profound and sustained hypotension. The technique is therefore unsuitable for routine clinical use [24]. Hypotension during surgery is the main risk factor for POCD because it can lead to cerebral hypoperfusion and decreased cerebral flow [25]. Therefore, it is crucial to optimize the profiles of dexmedetomidine administration to avoid undesirable haemodynamic side effects.

The common dosage of dexmedetomidine infusion during general anaesthesia in elderly patients undergoing major noncardiac surgery was shown in previous research: a loading dose of 0.5–1.0 μg/kg within 10–15 min or intraoperative continuous infusion at 0.2–0.7 μg/kg/h according to anaesthesia system records [26]. In this study, dexmedetomidine was administered at different loading doses (0.25 to 0.75 μg/kg within 15 min) followed by a fixed maintenance dosage (0.5 μg/kg/h). Our results showed that the higher loading doses of dexmedetomidine (0.5–0.75 μg/kg within 15 min) always resulted in more significant inhibition of MAP and HR. This results in haemodynamic changes of dexmedetomidine mainly towards hypotension and bradycardia. In fact, 14 patients in the D0.75 group withdrew from the study due to persistent and irreversible haemodynamic instability. In addition, the frequency and dose of vasoactive drug application in the high-dose dexmedetomidine group were generally increased. Bradycardia caused by dexmedetomidine is common in elderly individuals. There is a clear dose–response relationship between the infusion management of dexmedetomidine and a decrease in heart rate, whether at low or high dosages [27]. Patients administered 0.5 μg/kg/h dexmedetomidine intravenously from anaesthesia induction until chest closure had an increased incidence of bradycardia [11]. Low-dose dexmedetomidine infusion (0.5 μg/kg/h) was also found to cause bradycardia in elderly male patients within 7 days following thoracoscopic lobectomy [11].

Dexmedetomidine has typical biphasic haemodynamic effects, including transient hypertension, bradycardia and hypotension [27]. This was explained by the results from the peripheral vasoconstrictive and sympatholytic properties of dexmedetomidine [27, 28]. The sympatholytic effect of dexmedetomidine predominates at low plasma concentrations, presenting as lower MAP and heart rate. Vasoconstriction and bradycardia can commonly occur early after infusion of this drug, and hypotension can easily develop once sympathetic nervous activity is suppressed [27]. This effect is mediated by activation of presynaptic α2-adrenoceptors in the central nervous system and activation of α2-adrenoceptors in vascular endothelial cells, which causes vasodilation [27]. The researchers found that the application of dexmedetomidine at low dosages (0.25 to 1.0 mg/kg over 5 min) in healthy young volunteers could lead to a decrease in MAP levels of approximately 25–27% below baseline, even at increasing doses; the recovery period (time to return to baseline MAP once the infusion is stopped) increased from 3.7 to 13 h for the 0.25 and 4.0 mg/kg doses, respectively; but if dexmedetomidine was administered at higher doses (2 and 4 mg/kg over 5 min), profound hypertension appeared [27].

Emergence agitation is also common in adult patients after general anaesthesia [20, 29]. Several studies have shown that dexmedetomidine is effective in reducing emergence agitation [6, 30]. Dexmedetomidine 0.6 μg/kg before the induction of general anaesthesia, followed by dexmedetomidine 0.4 μg/kg/h until peritoneal closure, could reduce the incidences of agitation to the patients who undergoing elective open gastrectomy under TIVA general anaesthesia [6]. Intraoperative infusion of dexmedetomidine at a rate of 0.4 μg/kg/h from induction of anaesthesia until extubation provided smooth emergence and improved quality of recovery after nasal surgery [30]. Consistent with previous results, we found that during the post-anaesthesia recovery period, dexmedetomidine (D0.5 and D0.75 groups) significantly improved tolerance to endotracheal tubes and alleviated recovery agitation. Moreover, the higher the dose of dexmedetomidine was, the more stable the recovery. In addition, the haemodynamics of the recovery period were more stable and the recovery situation was more comfortable. These advantages may be beneficial to the postoperative recovery stage of patients. The sedation effect of dexmedetomidine is dose dependent, and high dosages could lead to sedation that is too deep for easy recovery. In the current study, the application of dexmedetomidine in elderly patients under general anaesthesia did not prolong the recovery time, with loading doses of 0.25–0.75 μg/kg for 15 min followed by a continuous infusion of 0.5 μg/kg/h until the end of the operation. Except for haemodynamic changes during the operation, no other adverse reactions were found in this study. Postoperative VAS scores showed that dexmedetomidine could alleviate postoperative pain effectively in a dose-dependent manner.

## Limitations

The present study had several limitations. First, the patients’ cognitive status could not be directly evaluated. The patients were formally assessed for POD only when they outwardly manifested obvious abnormal behaviours, and the incidence of POD might be underestimated. Second, except for CRP, biomarkers of inflammation were not measured in our study, and it is not possible to assess whether dexmedetomidine could suppress the inflammation caused by surgical stress. Third, the amount of blood loss, blood transfusion, and the frequency and dose of vasoactive drug administration in each patient were not recorded and statistically analysed. These issues need to be explored in future studies.

## Conclusion

In this study, we compared the effects of dexmedetomidine on haemodynamics and postoperative recovery in elderly patients undergoing hip replacement with different initial loading doses (0.25/0.5/0.75 μg/kg infused for 15 min) combined with continuous infusion of 0.5 μg/kg/h. In summary, dexmedetomidine could alleviate the agitation of elderly patients undergoing hip replacement after intravenous general anaesthesia combined with inhaled sevoflurane, and there was no delayed awakening from general anaesthesia. However, it is necessary to be vigilant about the haemodynamic inhibition of the drug at high dosages throughout the perioperative period. Dexmedetomidine 0.25–0.5 μg/kg as the initial loading dose followed by 0.5 μg/kg/h continuous infusion might provide comfortable recovery after general anaesthesia with slight haemodynamic inhibition.

## Data Availability

The datasets used and/or analysed during the current study are available from the corresponding author on reasonable request.
